# Integrated Team‐Based Learning in a UK Undergraduate Medical Programme

**DOI:** 10.1111/tct.70470

**Published:** 2026-07-03

**Authors:** Aaron Drovandi, Elizabeth Sheader, Margaret Kingston

**Affiliations:** ^1^ School of Medical Sciences, Faculty of Biology, Medicine and Health University of Manchester Manchester UK

**Keywords:** medicine, mixed‐methods, pedagogy, tertiary education

## Abstract

**Background:**

There is limited published evidence supporting integrated team‐based learning (TBL) as an effective method for teaching undergraduate medical students. This study describes student and staff perceptions, assessment outcomes and financial factors after integrated TBL was implemented into Year 1 of a large UK undergraduate medical programme.

**Methods:**

Five methods of data collection were used. An online survey was distributed to students, focus groups held with academic and technical staff, observation of teaching sessions, analysis of student assessment data and calculation of expenses for TBL delivery. Quantitative data were summarised narratively; qualitative survey data were analysed using conceptual content analysis; focus group data were analysed using inductive thematic analysis; and expenses data were summarised narratively and compared with problem‐based learning (PBL).

**Results:**

A total of 449 participants were involved in this study. Students and staff had overall positive perceptions of TBL, highlighting the engaging and consistent teaching and learning approach, effective teamworking and real‐world applicability of the weekly themes. Limitations raised were focused on logistical issues such as using new technology and session timing. Compared with previous cohorts taught through PBL, assessment analysis found mixed results by assessment type. Finally, TBL was found to be more financially viable than PBL through reduced staff time requirements despite initial cost outlays.

**Conclusion:**

TBL represents a potentially effective and efficient method for teaching undergraduate medical students on a large scale and should be considered by other medical programmes where increasing student numbers may affect the quality of PBL teaching.

**Abbreviations**
AES: application exercises
CCA: clinical competency assessment
CHERRIES: Checklist for Reporting Results of Internet E‐Surveys
COREQ: Consolidated criteria for Reporting Qualitative research
ILOs: intended learning outcomes
IQR: interquartile range
IRAT: individual readiness assurance test
LAMS: Learning Activity Management System
MCQ: multiple‐choice questionnaire
PBL: problem‐based learning
SD: standard deviation
TBL: team‐based learning
TRAT: team readiness assurance test

## 1 Introduction

Problem‐based learning (PBL) is a common pedagogical approach in undergraduate medical education globally, having been used for over 40 years [1, 2]. The move from ‘conventional’ didactic teaching to PBL is considered a major milestone in medical education, particularly relating to student development of critical thinking, student engagement and satisfaction [3, 4, 5]. However, PBL has several limitations which may limit its feasibility and effectiveness. PBL can be resource‐intensive, especially for large cohorts, and it experiences difficulties with standardising the student learning experience, poor or variable student engagement, limited coverage of content and issues with student team dynamics [6, 7, 8, 9, 10].

Team‐based learning (TBL) is an alternative pedagogical approach sharing many similarities with PBL [11]. These include having a student‐centred approach with active participation and emphasis on self‐directed learning, students learning in groups and centring learning around themes on a specific disease or body system [11, 12, 13, 14]. Key differences relate to having multiple smaller groups working in a room facilitated by several academics, in a more structured and facilitator‐guided learning environment. Furthermore, TBL uses individual and team readiness assurance tests (IRAT and TRAT) to support students in gauging the breadth and depth of their knowledge, supported by immediate feedback and discussion [11, 12, 13, 14].

*TBL uses individual and team readiness assurance tests (IRAT and TRAT) to support students in gauging the breadth and depth of their knowledge, supported by immediate feedback and discussion*.

TBL has been implemented widely in academia at the modular or sub‐modular level within multiple healthcare professional degrees (such as pharmacy, nursing and dentistry) as well as at programme level for some of these degrees [15, 16, 17, 18, 19]. However, this is not the case for medicine, with limited published evidence on the effectiveness of TBL in medicine when implemented at an integrated programme level, such as done at the University of Sydney [12, 13, 14, 20, 21]. This study therefore aims to build on the evolving literature surrounding TBL as a pedagogical approach when implemented at an integrated programme level for undergraduate medical students. The primary research question for this study was ‘How do medical students and academics perceive TBL as an effective pedagogical approach, including in comparison to PBL?’

### 1.1 The University of Manchester Medical Programme

Until recently, the University of Manchester utilised PBL as its main teaching approach through weekly cycles [22], firstly involving a 1‐h ‘opening session’ where student groups reviewed and identified important elements within a case and designed their own learning agenda to meet weekly intended learning outcomes (ILOs). Students are then given a range of teaching activities (e.g., lectures, dissection room) on associated clinical and non‐clinical topics. Finally, during a 1.5‐h ‘closing session’, student groups discuss answers to their learning agenda questions to ensure each ILO is addressed. This discussion is led by a student chairperson and supported by an academic facilitator. Students are assessed through multiple‐choice questionnaire (MCQ) semester tests (year‐specific), progress tests (consistent questions for all five year levels) and clinical competency assessments (CCAs [elsewhere referred to as OSCEs]). The programme is amongst the largest in the country, enrolling approximately 450 Year 1 students annually.

**FIGURE 1:**
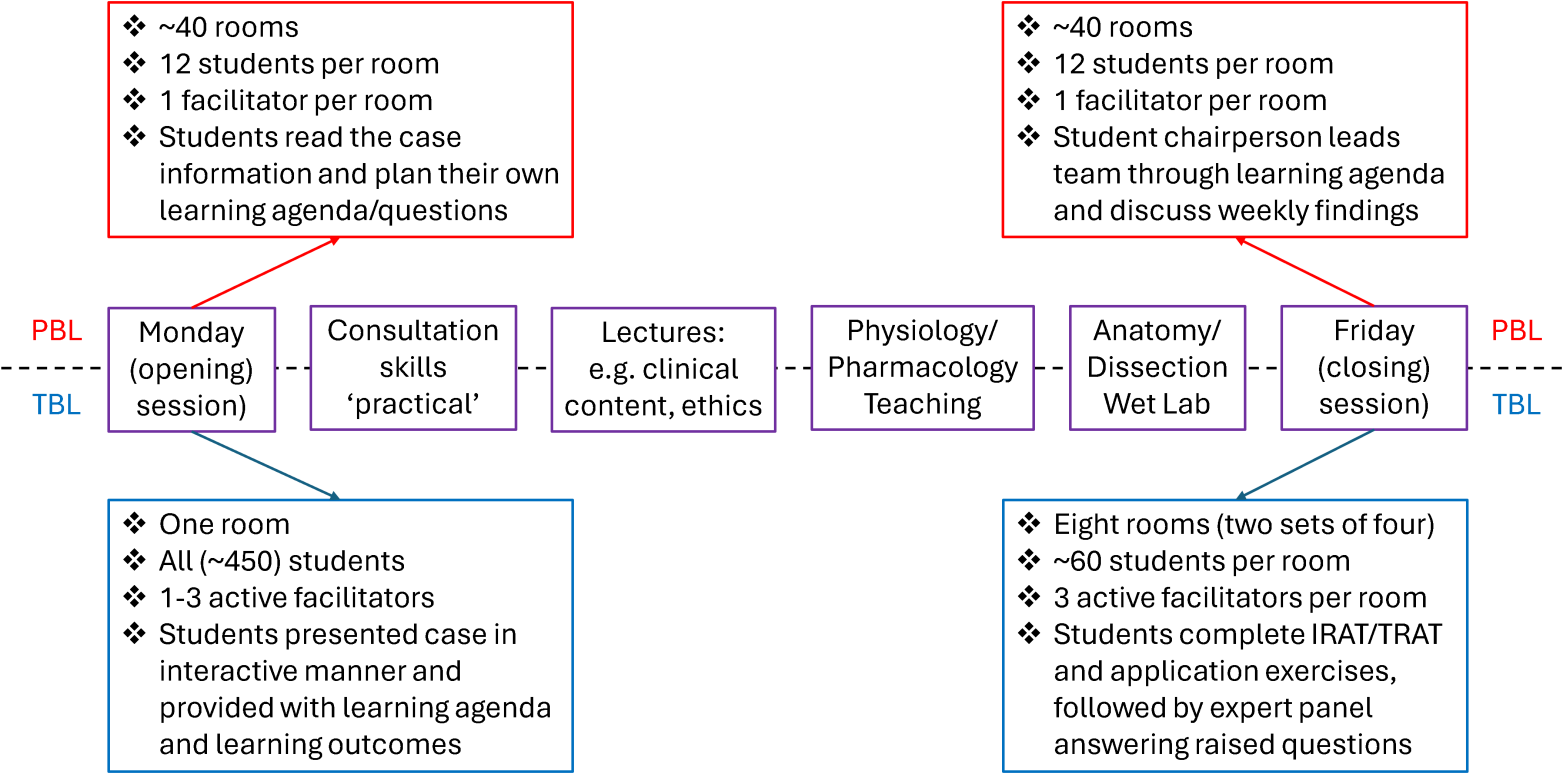
Diagram of a typical weekly theme, comparing TBL to PBL.

Commencing for the 2023/2024 academic year, the University of Manchester MBChB became one of few globally to use an integrated TBL approach for their undergraduate medical programme. Within this context, integrated refers to TBL being the central approach around which all teaching activities are delivered for the entire academic year. Description of how TBL was implemented aims to align to the guideline published by Haidet et al. on reporting TBL for health sciences education to ensure transparency in approach and confidence in interpretation [23]. Similar to how PBL was implemented, TBL is delivered in weekly cycles, commencing with a 1‐h ‘opening’ session involving the whole cohort attending a single interactive lecture theatre event, with two to three academics presenting core information through simulation, narrated roleplay or interactive exercises. The learning agenda is also provided and discussed alongside provision of ILOs. Mid‐week teaching activities are then provided as per the PBL approach, followed by a 2‐h ‘closing’ session involving students working in teams of six, in a larger learning community of 8–10 teams per room. Figure 1 outlines the broad structure of TBL, including similarities and differences to PBL.

For the ‘closing’ sessions, due to the size of the cohort, four TBL rooms are active simultaneously, each facilitated by three academics and digitally interconnected by a central coordinator who manages the timing of activities. Team members are allocated randomly to their team of six, with gender being the only controlled factor ensuring an even split of male and female students. TBL closing sessions are divided into; closed‐book IRATs and TRATs with discussion and feedback (30 min), application exercises (AES) with discussion and feedback (60 min) and an expert panel discussion (30 min). The IRAT, TRAT and AES are delivered using an online platform (Learning Activity Management System; LAMS) which allows facilitators to track student progress in real‐time, including identifying questions or concepts the cohort struggled with. Students have individual log‐in details and answered the 10 IRAT questions independently on their personal devices, with a team leader chosen each week to enter the teams' 10 TRAT and AES responses. During AES student teams apply knowledge to clinical scenarios or questions, utilising digital resources such as diagrams or clinical images, followed by room‐wide discussions. IRAT, TRAT and AES responses are formative only and do not contribute to grades. Lastly, the expert panel, composed of basic science and clinical specialists, answer ‘burning questions’ raised which could be submitted by students using LAMS as well as concepts the cohort struggled with. Finally, twice per semester, students complete formative peer review feedback of each other's contributions.

## 2 Methods

This study employed a mixed‐methods approach to answer the research question, including surveys with students, focus groups with academic and technical staff, observations of teaching sessions, and analysis of assessment and financial expenses data. Ethical approval was granted by the University of Manchester Proportionate Research Ethics Committee (2024‐18448‐32454). This study is reported according to the Consolidated criteria for Reporting Qualitative research (COREQ) and Checklist for Reporting Results of Internet E‐Surveys (CHERRIES) guidelines [24, 25]. All participants gave written informed consent before participating in the study.

### 2.1 Participants and Recruitment

All Year 1 undergraduate medical students enrolled during Semester 2 of the 2023/2024 academic year (*n* = 434) were invited to participate in the survey. This allowed students to overcome difficulties associated with the initial transition into medical school and provide better insights into their experiences associated with learning via TBL [26]. All academic staff involved in the development and/or facilitation of TBL sessions (*n* = 28) were invited to participate in a focus group, as were technical staff responsible for LAMS (*n* = 2).

### 2.2 Study Design and Data Collection

The survey was developed by the TBL academic team and senior academic leadership, and it was informed by previously validated research that focussed on team dynamics, student preparedness, and tutor guidance and provision of feedback [14]. The full survey is available in Appendix S1 (with comparisons to previous research in Appendix S2), which asked students 5‐point Likert scale and open‐ended questions on their perceptions of the overall TBL experience, positive and negative elements, resources provided, teaching session structure, content and delivery, functionality of their team, support and facilitation from academics and clinicians, and their perceived ease of learning the required materials. The survey link (via Mentimeter, an online interactive tool) was emailed each week to a randomised sub‐cohort to ensure all themes received feedback without overloading students with repeated requests for responses. The use of unique students' numbers prevented students from receiving multiple requests and providing multiple responses to the survey. The survey was reviewed and approved by the medical programme and TBL leadership teams, though was not pilot tested with students. Semi‐structured focus groups with academic and technical staff were developed and conducted by the first author, an experienced qualitative researcher (BPharm, MPharmPH, PhD) (see Appendices S3–S5). All three focus groups were scheduled for a maximum of 60 min in April 2024 and were audio recorded. Session observations were conducted by the first author, who observed each room twice during the semester. Student assessment data and financial expenses data were provided by the medical school examination team and senior leadership, respectively.

### 2.3 Data Analysis

Quantitative data were analysed using SPSS v29 (IBM Corp, Armonk, NY, USA). Participant characteristics Likert‐scale ratings were summarised narratively and compared between participant gender, age, ethnicity and origin using the Mann–Whitney U nonparametric test. Bonferroni adjustments were used in determining statistically significant differences between categories. Open‐ended responses were analysed using conceptual content analysis to compare emerging themes to Likert‐scale questions [27]. This involved identification, coding and quantification of key concepts raised relevant to its partner quantitative question, which was performed by the first author, followed by meetings and arriving at consensus with the co‐authors. Illustrative quotes that demonstrate the key themes identified are reported verbatim to support the findings. Focus group recordings were transcribed verbatim and thematically analysed as advocated by Braun and Clarke, using NVivo v12 (QSR International Pty Ltd) [28]. The first author familiarised themselves with the transcripts and independently coded using a line‐by‐line open coding process, with themes identified using a constant comparison process as advocated by Corbin and Strauss [29]. A provisional codebook was developed after the first focus group was analysed, iteratively refined as additional transcripts were analysed, and then finally compared with the initial transcript. Generated themes were reviewed to confirm points of data convergence and reach consensus with the other authors for points of data divergence. The themes generated were sent to participants by email for member checking, which did not receive responses relevant to the research question. Assessment data were tabulated and compared between participant characteristics (gender, age, ethnicity and origin) using the Mann–Whitney U nonparametric test. Comparisons were also made between TBL cohort data and the two previous years of PBL cohort data using independent sample *t*‐tests. Financial expenses data were tabulated and summarised narratively, comparing costs for running the TBL and PBL pedagogical approaches at the University of Manchester.

## 3 Results

A total of 449 participants were involved, including assessment data from 433 students (three withdrew prior to exams), survey data from 253 (58%) students, focus group data from 14 (50%) of the academic staff and two (100%) of the technical staff. Student traits are summarised in Table 1. There were slightly more female students (244; 56%), with a median age of 19 years, from the United Kingdom (87.2%) and from a diverse range of ethnic backgrounds. Staff characteristics including their TBL roles and responsibilities are summarised in Appendix S6.

**TABLE 1:** Characteristics for students receiving TBL learning in 2023/2024 (*n* = 436).

Characteristic	Total cohort	Non‐participants^a^	Participants^a^
Gender			
Female	244 (56.0%)	100 (54.6%)	144 (56.9%)
Male	192 (44.0%)	83 (45.4%)	109 (43.1%)
Age			
Mean (SD)	19.5 (1.7)	19.6 (1.5)	19.5 (1.7)
Median (IQR)	19.0 (19–20)	19 (19–20)	19 (19–20)
Range	18–38	18–27	18–38
Origin			
UK student	380 (87.2%)	156 (85.2%)	224 (88.5%)
International student	56 (12.8%)	27 (14.8%)	29 (11.5%)
Ethnicity			
African	29 (6.7%)	14 (7.7%)	15 (5.9%)
Arab	69 (15.8%)	34 (18.6%)	35 (13.8%)
Bangladeshi	17 (3.9%)	9 (4.9%)	8 (3.2%)
Chinese	11 (2.5%)	5 (2.7%)	6 (2.4%)
Indian	77 (17.7%)	31 (16.9%)	46 (18.2%)
Other Asian heritage	25 (5.7%)	13 (7.1%)	12 (4.7%)
Pakistani	66 (15.1%)	32 (17.5%)	34 (13.4%)
White British	125 (28.7%)	36 (19.7%)	**89 (35.2%)** ***
Other backgrounds	17 (3.9%)	9 (4.9%)	8 (3.2%)
Examination attainment			
Semester Test 1 %	63.8 (9.9)	62.8 (10.7)	64.6 (9.1)
Semester Test 2 %	61.6 (10.1)	60.8 (9.4)	62.3 (10.6)
Progress Test 1 %	30.3 (5.6)	29.9 (5.9)	30.6 (5.4)
Progress Test 2 %	36.3 (6.8)	35.5 (6.3)	**36.9 (7.1)** *
CCA stations passed	8.0 (1.6)	7.9 (1.6)	8.1 (1.6)
CCA stations excelled	3.3 (1.9)	3.0 (1.7)	**3.5 (2.0)** *

^a^ Refers to those who did not participate, and those who did participate in the online survey.

* *p* < 0.05.

** *p* < 0.01.

*** *p* < 0.001.

### 3.1 Student Survey

A summary of Likert‐scale responses is detailed in Appendix S7. Overall, students had positive perceptions of TBL, with nearly three‐quarters (184; 72.7%) having a ‘positive’ or ‘very positive’ experience. Other elements positively received (≥ 75% ‘positive’ or ‘very positive’) included the ease of accessing learning resources (Q7), clearly defined ILOs (Q8), interactive and engaging TBL sessions (Q14), team composition, participation, effective communication, disagreement resolution and equal voice and respect (Q15–Q19), facilitator knowledge and support (Q21, Q23), variety of weekly teaching sessions (Q24) and real‐world applicability of the weekly themes (Q26). None of the Likert‐scales received excessively negative responses (≥ 20% ‘very negative’ or ‘negative’). Female students were more negative about their overall TBL experience (*p* = 0.004), and ‘non‐White’ students had more positive perceptions of TBL overall (*p* = 0.008), resource relevance to sessions (*p* = 0.001), clarity of requirements before attending the Friday session (*p* = 0.007), the value of varied teaching sessions (*p* = 0.003) and understanding materials to the depth required (*p* < 0.001).

Themes from the open‐ended questions reinforced Likert‐scale responses. Additional quotes to those included below illustrating themes are available in Appendix S8. The variety of learning activities offered each week, along with the engagement and clinical relevance of these, was the strongest theme raised (88; 34% of respondents). Conventional lectures, mini‐learning sessions, consultation skills sessions and TBL ‘closing’ sessions were all positively received.

The mini learning sessions were very short and engaging so it was easy to stay focused and also meant that I did not have to watch the recordings multiple times to understand.

The [closing] session was very helpful, especially as it was focused on drugs and side effects part of the case, which highlights the importance of knowing the mechanisms.

Within the closing session, the IRAT and AES were considered particularly beneficial by students (66; 26% of respondents) by allowing them to gauge their own breadth and depth of knowledge and to apply knowledge to scenarios perceived as pertinent to clinical practice.

I liked the TRAT discussion and found it to be useful for my learning as I could see where my team members knew the topics which I struggled with and vice versa.

Students also felt confident working in teams and communicating effectively to answer TRAT and AES, and they felt supported by facilitators who guided them through the content and activities.

Members of my TBL group are respectful, allowing everyone to feel comfortable and discuss their ideas and explanations, allows any disagreements to be resolved quickly.

Facilitators were great: they engaged actively with us and helped us to better understand topic by guiding us through our thought processes.

Logistical issues were the most frequently raised limitation. Students valued discussions for the IRAT/TRAT and AES and felt more time was needed to delve into questions and answers more comprehensively. They highlighted frustration when discussions were cut short by the central coordinator responsible for keeping rooms synchronised. From 51 students (21% of respondents) describing this, one student provided a response summarising this issue.

There were several times where the discussion in our room was interrupted by needing to move on to the next activity … it's been a recurring problem. I find it frustrating that we often get to the height of the discussion just before being cut off by having to move on with the other groups to the next exercise.

Timing issues were also raised regarding the expert panel, with students (36; 14% of respondents) wanting them to discuss more of the questions raised by the student cohort.

The expert panel in semester 2 has been really great and interactive! Sometimes, some burning questions … are not answered as we run out of time, could we prioritise them?

The experts were very useful as usual, but did not have time to answer questions that seemed quite important for the case which was a shame.

### 3.2 Staff Focus Groups and Session Observations

From the three staff focus groups and eight session observations, two broad‐level themes were generated: (1) student learning and facilitator roles, and (2) comparisons of TBL to PBL. These are described in Table 2, with their respective sub‐themes and quotes. For the first theme, as the purpose of implementing TBL was to improve the quality and consistency of student learning, all focus groups spoke about student learning outcomes, with three sub‐themes identified: (1) student preparedness for TBL sessions, (2) student engagement during TBL and (3) teamworking (as it applies to student learning). For the second theme, as most participants had several years of experience previously facilitating PBL groups, conversations around TBL naturally compared their experiences and perceptions between the two pedagogies. These discussions fell under four sub‐themes: (1) facilitator interactions with students, (2) facilitator teamworking, (3) effort and reward for facilitators and (4) overall student learning impacts.

**TABLE 2:** Sub‐themes from the focus groups and session observations, including key data and representative quotes.

Sub‐theme	Key data	Representative quote(s)
Theme 1: Preparedness	High but variable (especially around holidays); measured by facilitators using LAMS system; could affect the quality of sessions by replacing facilitating with teaching	‘That made it very difficult to have a group discussion … as it has gone on I have done more revision of the cardiovascular system which is not my expert topic just to be able to help facilitation’ (P14), ‘I have really felt the facilitators notes were really useful and very much a “this is not for us to teach if you do not know it, it is your responsibility to have been prepared”’ (P11)
Theme 1: Engagement	Opening sessions helped build a learning community; all elements of the closing sessions engaging for students to consolidate and apply weekly learning; expert panel needed to stay ‘on task’ to retain engagement; easier to identify unengaged students	‘There are about ten of us every Monday … it is really enjoyable, unless you have other things to do you make an effort to go, surely if we enjoy it the students will’ (P9), ‘Students are consistently quiet and focussed during the IRAT … the switch from IRAT to TRAT is amazing, the room always explodes into engaging conversation on every table’ (Observer), ‘I think they are engaged in the TRAT and the application exercises, but they do switch off during the expert panel [agreement from room], well some not all students’ (P8), ‘The smaller group sizes make it harder to hide, so you have to participate. The level of noise is quite active, active engagement noise … I can say I wasn't all for introducing TBL, but I actually think students have actively engaged better with it’ (P3)
Theme 1: Teamworking (on student learning)	Better teaching environment for staff; improved student relationship building and accountability	‘That enjoyment factor is there and I think the students can see it, the banter between the staff and passing tasks between colleagues, and we want them to see that we are a team and we are collegiate’ (P1), ‘They are obviously very conscious of their engagement with their team, which I think is really good that they have that teamworking’ (P11)
Theme 2: Interactions with students	Increased student exposure to academic team and experts compared with strong professional bonds to individual tutor; easier to identify and support students not engaging with the materials or their team; better consistency of student education at the expense of tailored conversations; better clinical application of cases	‘There is something nice about getting to know that group of students really well through the semester, you do not have that same sort of relationship’ (P6), ‘They are in four rooms, before we had forty rooms so I think there is definitely more consistency by the TBL format as opposed to PBL because they get the same ILO and learning agenda, and have discussions with the experts across all the rooms’ (P9), ‘I think the discussions we found can be cut quite short and are being rushed’ (P14), ‘It's one of the bits that are more engaging, when the clinicians are discussing “well this is how I manage this patient and this is what the guidelines say” … I think that is really useful getting some insight from clinicians [agreement from others]’ (P8)
Theme 2: Teamworking (facilitator roles)	Improved student confidence; better sense of community; collegiate rather than isolated working environment	‘I think it increase confidence in students by being in that smaller team being able to ask each other, and to be wrong and make mistakes and why that might not necessarily be the right answer, rather than in large groups saying “I do not really understand”’ (P11), ‘Coming into a room where students are quite avidly discussing a topic there is much more a sense of community that you did not really have with individual rooms with students in PBL, there was always a disconnect that I found as a tutor’ (P12), ‘In the [TBL] design team we had representatives from all of the components of teaching’ (P1), ‘we have closer links now to those groups, I would have not known a single person in the communications skills team prior starting TBL’ (P2)
Theme 2: Effort and reward	Required more effort to develop, organise and run for facilitators; increased satisfaction with teaching	‘With PBL you just turned up and had a discussion with your group’ (P10), ‘It is much more than being a facilitator, it is being a presenter, a game show host, you know you feel sometimes in a room of sixty you have to keep the energy going and I've noticed that it takes a lot more out of you’ (P1), ‘After ten years of PBL I feel a bit more invigorated … we actually have got people who want to teach not people who are forced to teach, that is a big difference’ (P3), ‘Working with this group of colleagues has rejuvenated my enjoyment of teaching’ (P2)
Theme 2: Overall student learning	Engagement and teamworking improvements improving overall experience	‘When that TRAT section starts and they all start discussing, that never happened in the PBL room … they never did this level of discussion’ (P4) ‘There is a massive improvement in the quality of the work when doing the application exercises … it's much more akin to the way students have learnt in their school and college experience so I think it is easier for them, so I think it has improved’ (P11)

Abbreviations: ILO: intended learning outcome; IRAT: individual readiness assurance test; PBL: problem‐based learning; TBL: team‐based learning; TRAT: team readiness assurance test.

### 3.3 Assessment Data

The mean (standard deviation; SD), median (interquartile range; IQR) and range of percentage scores for the two semester tests and two progress tests, as well as the number of CCA stations (out of 10) that were passed and the number marked as ‘excellent’ for the TBL cohort and two previous years of PBL cohorts are available in Table 3. Semester tests are specific to the content taught in each semester and include a similar proportion of questions for each topic area each year (e.g., anatomy). They are subject to content and difficulty modifications year‐on‐year according to changes in the programme. The TBL cohort (2023/2024 academic year) performed significantly better than the 2021/2022 PBL cohort, though significantly worse than the 2022/2023 PBL cohort for the Semester 1 Test, and significantly worse than both PBL cohorts for the Semester 2 tests (all *p* < 0.001). Progress tests by contrast are consistent across all five year‐levels with students in Year 1 being taught PBL or TBL being given the same questions as Year 5 students. The intention being to challenge students across much broader content knowledge, as well as demonstrate to students how their knowledge progresses year on year. For both progress tests, the TBL cohort performed significantly better compared with PBL cohorts (all *p* < 0.01). The three cohorts performed similarly in passing their CCA stations, though the TBL cohort performed significantly better in having stations marked as ‘excellent’ compared with PBL cohorts (both *p* < 0.05).

**TABLE 3:** Comparison of assessment outcomes for TBL cohort versus two previous PBL cohorts.

Assessment type	PBL 1 (2021/22)	PBL 2 (2022/23)	Combined PBL 1 + 2	TBL (2023/24)	Vs. PBL 1	Vs. PBL 2	Vs. both
	(*n* = 507)	(*n* = 416)	(*n* = 923)	(*n* = 433)	*p*	*p*	*p*
Semester Test 1							
Mean (SD)	61.71 (9.78)	68.73 (9.13)	64.88 (10.11)	63.80 (9.86)	< 0.001 (+)	< 0.001 (−)	0.067
Median (IQR)	61 (56–69)	69 (63–75)	66 (58–72)	65 (58–70)	< 0.001 (+)	< 0.001 (−)	0.067
Range	31–85	29–89	29–89	33–85			
Semester Test 2							
Mean (SD)	66.44 (10.79)	70.86 (11.12)	68.43 (11.19)	61.59 (10.15)	< 0.001 (−)	< 0.001 (−)	< 0.001 (−)
Median (IQR)	66 (59–75)	72 (63–79)	69 (61–77)	62 (54–69)	< 0.001 (−)	< 0.001 (−)	< 0.001 (−)
Range	55–91	34–93	34–93	23–85			
Progress Test 1							
Mean (SD)	29.10 (7.76)	28.44 (5.02)	28.80 (6.66)	30.29 (5.63)	0.009 (+)	< 0.001 (+)	< 0.001 (+)
Median (IQR)	28 (25–32)	28 (25–31)	28 (25–31)	30 (26–34)	< 0.001 (+)	< 0.001 (+)	< 0.001 (+)
Range	17–77	17–48	17–77	Oct‐65			
Progress Test 2							
Mean (SD)	27.57 (5.59)	30.86 (7.53)	29.05 (6.73)	36.27 (6.84)	< 0.001 (+)	< 0.001 (+)	< 0.001 (+)
Median (IQR)	27 (24–31)	31 (26–35)	29 (24–33)	36 (31–41)	< 0.001 (+)	< 0.001 (+)	< 0.001 (+)
Range	14–46	16–60	14–60	21–71			
CCA (10 stations)							
Passed	8 (7–9)	8 (7–9)	8 (7–9)	8 (7–9)	0.656	0.119	0.583
Excellent	3 (2–4)	3 (1–4)	3 (2–4)	3 (2–5)	0.041 (+)	0.021 (+)	0.013 (+)

*Note:* Means compared using independent samples *t*‐test. Medians compared using Mann–Whitney U test. (−) and (+) indicates if the TBL cohort performed significantly worse and significantly better, respectively, compared with the PBL cohort(s).

Abbreviations: PBL: problem‐based learning; TBL: team‐based learning.

### 3.4 Financial Expenses Data

The comparative financial costs of running TBL to PBL are available in Appendix S9, considering facilitator preparation and facilitation time, learning technology costs and e‐Learning technologist support. According to these costs, TBL is slightly more cost effective than PBL despite the initial outlay required for technological devices to support room‐to‐room videoconferencing. These costs would be distributed across several years of use, further offset by the fact that most of the facilitators facilitated both the morning and afternoon sessions, reducing ‘preparation time’ required. Not included in these calculations is the staff time required to design the TBL sessions or to revise and update PBL sessions year on year.

## 4 Discussion

This study investigated TBL as a pedagogical approach for undergraduate medical students on a large scale, including perceptions and potential effectiveness compared with the previously used PBL approach, using a mixed‐methods approach with five data sources. It was found that students and staff both had positive perceptions of TBL, through providing students with a structured, engaging and consistent learning environment, that simultaneously provided staff with a more enjoyable and collaborative teaching experience. Compared with PBL, the use of more interactive opening sessions, as well as IRAT, TRAT, AES and expert panel discussions during the closing sessions provided students with opportunities to gauge and apply their knowledge for a similar financial cost to the medical school. Finally, assessment analyses had mixed results, with TBL students performing worse in semester tests, though better in progress tests.

The student transition into university is challenging, particularly when factors such as international travel, language barriers and financial strains are present, requiring universities to employ a range of supportive systems to ensure student wellbeing [26]. Compounding this are the difficulties associated with unknown or unrealistic expectations of teaching, learning and assessment in higher education [30]. In comparison to PBL, TBL may be more palatable to new learners at university by providing a more structured, consistent and engaging learning environment, aligning more closely with their experiences and expectations at high school or college [13, 30]. This may or may not be the case for mature‐aged students who have either not been formal learners for many years or have previously learned through traditional didactic methods in a university setting [26]. Introverted students and female students, particularly those from cultural backgrounds where women's voices are traditionally not equally heard to men's, may also have a varied experience with TBL and the expectation to verbally contribute in front of a room full of other students. Differences in gender‐based preferences and outcomes are not limited to TBL, with other pedagogies such as PBL experiencing this phenomenon [31, 32, 33].

*TBL may be more palatable to new learners at university by providing a more structured, consistent and engaging learning environment*.

In addition to TBL structure and consistency, the testing and application of knowledge to clinically relevant scenarios, supported by expert discussion and monitored through a centralised learning system, ensure student progress is maintained, and support and guidance are provided to students struggling to achieve the minimum threshold [34]. The downside of this consistency was a lack of individuality for groups and the frustration of students with having discussions cut short; however, it does further demonstrate student engagement with TBL IRAT/TRAT/AES and their desire to interact with each other and facilitators on key topics. Finally, TBL opening and closing sessions fostered larger scale learning environments and ‘communities of practice’, increasing student sense of belonging and development of their professional identity [35, 36]. Development of these is linked to more positive learning environments, as well as reduced burnout and recruitment retention, which are essential for students entering workplaces already burdened by these issues [37].

Each of these considerations, as well as positive student responses to the survey suggest that both overall and in comparison to PBL, for Year 1 undergraduate medical students, TBL has the potential to offer a high quality and satisfactory learning experience [38]. Whilst it could be expected that these positive perceptions across students and educators might translate to improved assessment outcomes compared with PBL‐taught cohorts [18, 39], there were inconclusive findings from the assessment analysis. The survey findings mirror many of those from the University of Sydney, where students preferred TBL over PBL due to the more structured and engaging learning approach, the IRAT/TRAT followed by facilitator‐led discussions, and resource efficiencies afforded through TBL [14, 20]. This study found these benefits within both a different geographical context (United Kingdom vs. Australia) and at a larger scale (~450 vs. ~280 students), with the use of LAMS and expert panel elements both positive novel additions. The value of the staff experience in facilitating TBL compared with PBL was also a key finding. Academia is experiencing large scale loss of talent to industry due to multiple issues including poor job satisfaction [40]. TBL provided academic staff with a more engaging and enjoyable teaching role, with the team‐working, collaboration and networking element of TBL positively received over the absence of these opportunities with PBL [41].

*TBL provided academic staff with a more engaging and enjoyable teaching role*.

A key strength of this study is the high proportion of students and staff participation in the survey and focus groups, respectively, and the variety of data collection sources used with triangulation identifying points of data convergence and divergence. The use of multiple assessment types as indicators of student performance further contributes to the robustness of the findings. Limitations of note include the unknown impact of COVID on the three cohorts being compared, each of which experienced the learning and social consequences of COVID lockdowns at different stages of their pre‐university education. Comparison across cohorts instead of within cohorts also inherently reduces certainty of the findings as cohorts may have inherent differences relating to cohort composition and broader contextual influences. Secondly, as this was the first time in which TBL was conducted in the University of Manchester MBChB, there may be aspects not reflective of how TBL would run in future academic years, including the novelty of TBL and greater engagement of staff involved in this first rollout, as well as challenges associated with logistical implementation and communication with students. Thirdly are the roles of the authors (each of whom holds senior positions in the medical programme) and their consequential interpretation of the data. However, this was mitigated through data collection and analysis being led by the first author who was not involved in the development or delivery of TBL, nor in the teaching of students in the programme. Fourthly, this study was conducted at a singular UK medical school reducing confidence in the generalisability of findings outside of this setting. Lastly, focus groups were planned with students though were not able to run due to time constraints, limiting the depth of the student voice originally planned, which may partially be mitigated through previous research whose findings align with those raised in this study relating to student perceptions of the value of the IRAT/TRAT, provision of immediate feedback, the engaging approach to learning and accountability to their team [12, 42].

## 5 Conclusions

TBL was positively received by both staff and students, resulted in higher assessment scores for some assessment types, and was financially viable in comparison to PBL for first year students at a large UK medical school. Limitations were mainly logistical in nature and are likely to improve after this first iteration. Future research requires long‐term follow‐up of students taught by TBL to understand their confidence and competence during the clinical years of their learning, as well as their effectiveness as practising medical professionals after graduation.

## Author Contributions

**Aaron Drovandi:** conceptualization, methodology, investigation, data curation, formal analysis, writing (original draft) and writing (review and editing). **Elizabeth Sheader:** conceptualization, writing (review and editing). **Margaret Kingston:** conceptualization, writing (review and editing).

## Funding

The authors have nothing to report.

## Ethics Statement

Ethical approval was granted by the University of Manchester Proportionate Research Ethics Committee (2024‐18448‐32454).

## Consent

Participants in the survey and focus groups gave written informed consent. Ethical approval for use of assessment data was waived.

## Conflicts of Interest

All authors are employees of the University of Manchester and have senior roles in the medical programme.

## Supporting information


**Appendix S1:** Student survey.
**Appendix S2:** Comparison of survey questions to those used in previous research.
**Appendix S3:** TBL ‘designer’ focus group.
**Appendix S4:** TBL facilitator focus group.
**Appendix S5:** Technical staff focus group.
**Appendix S6:** Academic and technical staff roles.
**Appendix S7:** Student responses to the 5‐point Likert scale survey questions (*n* = 253).
**Appendix S8:** Additional responses to open‐ended questions.
**Appendix S9:** Comparison of financial elements of PBL and TBL for one academic year.

## Data Availability

Data are available from the corresponding author upon reasonable request.
