# Supplementary material for: Integrated Team‐Based Learning in a UK Undergraduate Medical Programme
Source: Clin Teach. 2026 Jul 3;23(4):e70470. doi: 10.1111/tct.70470 (PMC13332307; doi:10.1111/tct.70470)
Supplement: Supplementary file 1 — Appendix S1: Student survey. Appendix S2: Comparison of survey questions to those used in previous research. Appendix S3: TBL ‘designer’ focus group. Appendix S4: TBL facilitator focus group. Appendix S5: Technical staff focus group. Appendix S6: Academic and technical staff roles. Appendix S7: Student responses to the 5‐point Likert scale survey questions (n = 253). Appendix S8: Additional responses to open‐ended questions. Appendix S9: Comparison of financial elements of PBL and TBL for one academic year. [file TCT-23-e70470-s001.docx]

Appendix 1 – Student Survey

1. **Overall Experience:**
   1. How would you rate your overall experience of the TBL case this week? (5-point scale from 1 = very negative to 5 = very positive)
   2. What were the aspect(s) of this TBL case and associated learning activities and resources that you enjoyed the most, or found the most useful? (open-ended; free-text response)
   3. What were the aspect(s) of this TBL case and associated learning activities and resources that you enjoyed the least, or found the least useful? (open-ended; free-text response)
   4. Do you feel that anything is missing from the overall TBL case experience that would make the process more conducive to your learning? (open-ended; free-text response)
2. **Pre-Friday-Session:** (5-point scales are from 1 = strongly disagree to 5 = strongly agree)
   1. The case was presented clearly and linked well with the mid-week learning activities and resources. (5-point scale)
   2. The resources required for the case were made clear to me. (5-point scale)
   3. The resources all seemed relevant to the session. (5-point scale)
   4. The resources were of an appropriate length (didn't take up too much time). (5-point scale)
   5. It was clear what needed to be done before the Friday session. (5-point scale)
   6. The resources were easy to access and use. (5-point scale)
   7. Is there anything else related to the previous 'Pre-Friday Session' questions you would like to highlight? (open-ended; free-text response)
3. **Session Structure and Delivery:** (5-point scales are from 1 = strongly disagree to 5 = strongly agree)
   1. The intended learning objectives were clearly defined at the start of the week. (5-point scale)
   2. The intended learning objectives were relevant to the content of the learning materials and the Friday session. (5-point scale)
   3. The content of the Friday session were worked through at an appropriate pace. (5-point scale)
   4. There was sufficient time available for questions and discussions. (5-point scale)
   5. The Monday and Friday sessions integrated well with each other and the other materials delivered during the week. (5-point scale)
   6. The sessions across the week were engaging, making attendance feel worthwhile. (5-point scale)
   7. Was the Friday TBL session engaging? (5-point scale)
   8. Is there anything related to the previous 'Session Structure and Delivery' questions you would like to highlight? (open-ended; free-text response)
4. **Team Functionality:** (5-point scales are from 1 = strongly disagree to 5 = strongly agree)
   1. My team has a good mix of students from different backgrounds, bringing in a range of different viewpoints. (5-point scale)
   2. Everyone in my team participates at a sufficient level. (5-point scale)
   3. There is effective communication between all of my team members. (5-point scale)
   4. Disagreements within my team are resolved effectively. (5-point scale)
   5. All team members are treated with equal respect and are given equal voice during the sessions. (5-point scale)
   6. Members of my team experience anxiety due to needing to work in a team environment. (5-point scale)
   7. Is there anything else related to the previous 'Team Functionality' questions you would like to highlight? (open-ended; free-text response)
5. **Academic and Clinical Input:** (5-point scales are from 1 = strongly disagree to 5 = strongly agree)
   1. The facilitators were knowledgeable about the topic and provided key information when it was needed. (5-point scale)
   2. The experts who Zoomed in to the session were effective in answering questions raised by the students. (5-point scale)
   3. The facilitators were physically available to provide us with guidance when it was needed. (5-point scale)
   4. Is there anything related to the previous 'Academic and Clinical Input' questions you would like to highlight? (open-ended; free-text response)
6. **Ease of Learning:** (5-point scales are from 1 = strongly disagree to 5 = strongly agree)
   1. The range of teaching sessions during the week helped in learning the materials related to the TBL theme. (5-point scale)
   2. I feel that I understood the overall case materials to the depth that the teaching staff expect. (5-point scale)
   3. What I learned during this week is applicable to real-world situations. (5-point scale)
   4. Is there anything related to the previous Ease of Learning' questions you would like to highlight? (open-ended; free-text response)

Appendix 2. Comparison of Survey Questions to those used in previous research.

| **Questions used in Burgess et al 2017** | **Questions used in the current study** |
| --- | --- |
| **Theme: Team Dynamics** | |
| All team members made an effort to participate in discussion | Replaced with “Everyone in my team participates at a sufficient level” |
| All team members consistently paid attention |  |
| Different points of view were respected by team members | Modified to “All team members are treated with equal respect and are given equal voice during the sessions” |
| Team members encouraged one another to express their opinions | Replaced with “There is effective communication between all of my team members” and “Disagreements within my team are resolved effectively” |
| My team actively elicited multiple points of view before deciding on a final answer |  |
| Team members used feedback about individual or team performance | Not used |
| The number of group members enhanced my experience of peer learning | Replaced with “My team has a good mix of students from different backgrounds, bringing in a range of different viewpoints” |
| - | Members of my team experience anxiety due to needing to work in a team environment |
| **Theme: Student Preparedness and Perceptions of Materials** | |
| Students did the readings prior to session | These two questions were replaced with several questions expanding on preparedness to also include interpretation of expectations and perceptions of preparatory material:  “The case was presented clearly and linked well with the mid-week learning activities and resources”, “The resources required for the case were made clear to me”, “The resources all seemed relevant to the session”, “The resources were of an appropriate length (didn't take up too much time)”, “It was clear what needed to be done before the Friday session”, “The resources were easy to access and use”, “The intended learning objectives were clearly defined at the start of the week”, The intended learning objectives were relevant to the content of the learning materials and the Friday session” |
| Completion of the prescribed out-of-class preparation assisted in my learning |  |
| **Theme: Session Structure and Delivery** | |
| None | See Questions 3a to 3h in Appendix 1 above |
| **Theme: Ease of Learning** |  |
| None | See questions 6a to 6d in Appendix 1 above |
| **Theme: Facilitator Support** | |
| I received useful and timely feedback from the tutor | These two questions were replaced with three related questions about the academic facilitators (rather than tutors) and the expert panel: a. The facilitators were knowledgeable about the topic and provided key information when it was needed. (5-point scale)  b. The experts who Zoomed in to the session were effective in answering questions raised by the students. (5-point scale)  c. The facilitators were physically available to provide us with guidance when it was needed. (5-point scale) |
| The tutor helped to focus discussions and learning |  |
| Problem solving allowed me to develop my clinical reasoning skills | - |
| Competitiveness between groups enhanced my learning | - |
| The individual and team tests at the beginning of class assisted in my learning | - |
| **Theme: Overall perceptions** | |
| - | How would you rate your overall experience of the TBL case this week? |

Appendix 3 – TBL ‘Designer’ Focus Group

1. **Motivations:** What were the main motivations in the change from PBL to TBL? What were your initial thoughts regarding this change, and have your thoughts changed during the process of designing and implementing TBL?
2. **Design Considerations**: Throughout the process of designing TBL, what factors or sources influenced its design? What phases of design occurred from initial conception to implementation?
3. **Collaboration and Communication**: How was the team for TBL assembled, and what were the processes behind ensuring the team functioned effectively?
4. **Barriers**: What were or have been the major barriers which made the design and implementation of TBL more difficult than it should have been? Do you feel that there are any logistic limitations of the overall TBL process?
5. **Preparation and Training:** What training was received (if any) relating to designing and implanting TBL? Is there any training that you wish you had, or was more comprehensive?
6. **Overall Positives:** What do you consider are the highlights of student learning through team-based learning? What do you think students enjoy the most each week and why?
7. **Overall Negatives:** What do you consider the lowlights of student learning through team-based learning? What do you think students enjoy the least each week and why?
8. **Other Thoughts:** Is there anything else about team-based learning that we haven’t discussed that you feel would be useful to describe to the academic team involved in teaching?

Appendix 4 – TBL Facilitator Focus Group

1. **Student Engagement:** Do you feel that students are adequately engaged with the TBL sessions? Do they seem adequately prepared for the sessions? Are there any aspects of the sessions they feel more or less engaged with compared to other aspects of the sessions?
2. **Student Teamworking:** How well do the student teams function overall? Does there appear to be exclusion of team members? How could the teams function more effectively?
3. **Logistical Considerations:** Do you feel that there are any logistic limitations of the overall TBL process? Is the timing of the sessions appropriate?
4. **Preparation and Training:** Do you feel that you have received sufficient training and allowed enough preparation time to effectively facilitate TBL sessions? Are there any resources that might improve your ability to facilitate these sessions?
5. **Physical Resources and Technology:** Does the physical space, and resources such as chairs, tables, and technology feel adequate for conducting TBL sessions? Is there any resources which are present but not needed? Is there anything additional you would prefer to facilitate sessions?
6. **Overall Positives:** What do you consider are the highlights of student learning through team-based learning? What do you think students enjoy the most each week and why?
7. **Overall Negatives:** What do you consider the lowlights of student learning through team-based learning? What do you think students enjoy the least each week and why?
8. **Other Thoughts:** Is there anything else about team-based learning that we haven’t discussed that you feel would be useful to describe to the academic team involved in teaching?

Appendix 5 – Technical Staff Focus Group

1. **Roles and Responsibilities**: Can you describe your responsibilities and tasks related to TBL and the use of the LAMS system? For comparison, did you have any roles or responsibilities relating to PBL?
2. **LAMS Functionality**: Do you feel that the LAMS system was able to effectively deliver what was required for TBL? Were there any elements that had to be modified to be deliverable by LAMS?
3. **LAMS Training**: As a new system, how did you find the quality of the training for LAMS, both for yourselves and the broader academic team involved in using LAMS? Was LAMS user friendly?
4. **Student Engagement with LAMS:** What were your perceptions of how well the students engaged with LAMS? Were there any consistent or significant issues experienced by the students?
5. **Barriers**: from your point of view were there any major barriers or limitations experienced during the implementation of TBL within the medical school?
6. **Supports**: what kind of supports did you receive in ensuring that TBL and the use of LAMS for TBL was successful? Were the physical resources including the rooms appropriate for TBL?
7. **Final comments**: Are there any other main positive or negatives of your experience with TBL and LAMS that we haven’t yet discussed?

Appendix 6 – Academic and Technical Staff Roles

| **ID** | Employed  Position | Years of Relevant Experience* | Role related to TBL | Years as a PBL facilitator |
| --- | --- | --- | --- | --- |
| **01** | Associate Professor (Physiology) | 25 years | Lead academic for TBL writing and implementation, and TBL facilitator | 20 years |
| **02** | Professor (Genetics) | 34 years | Curriculum planning group member and TBL facilitator | 20 years |
| **03** | Senior Lecturer (Genetics) | 25 years | Curriculum planning group member and TBL facilitator | 21 years |
| **04** | Senior Lecturer (Evolution, Infection and Genomics) | 22 years | Facilitator and TBL curriculum development | 22 years |
| **05** | Lecturer (Medical Biochemistry) | 4 years | Lead facilitator, e-learning liaison and implementation team member | 4 years |
| **06** | Senior Lecturer (Medical Education) | 20 years | Facilitator and TBL curriculum development | 20 years |
| **07** | Associate Professor (Anatomy) | 20 years | Semester lead and facilitator | 20 years |
| **08** | Lecturer (Diabetes & Endocrine) | 8 years | Lead facilitator, and TBL curriculum development | 2 years |
| **09** | Professor (Anatomy) | 23 years | Curriculum planning group member and TBL facilitator | 20 years |
| **10** | Senior Lecturer (Physiology/Pharmacology) | 20 years | Facilitator and TBL curriculum development | 20 years |
| **11** | Lecturer (Medical Education) | 11 years | Lead facilitator | 8 years |
| **12** | Lecturer (Cardiovascular Sci) | 10 years | Facilitator | 4 years |
| **13** | Clinical Teaching Fellow | 1 year | Facilitator | 1 year |
| **14** | Senior Lecturer (Biological Sciences) | 24 years | Facilitator | 14 years |
| **15** | e-Learning Technologist | 7 years | Lead e-Learning technologist | N/A |
| **16** | e-Learning Technologist | 8 years | Oversight of eLearning and support implementation | N/A |

* Relevant experience relates to experience in teaching students in a higher education setting.

Appendix 7. Student responses to the 5-point Likert scale survey questions (n=253).

|  | Very Negative | Negative | Neutral | Positive | Very Positive | Mean (SD) |
| --- | --- | --- | --- | --- | --- | --- |
| 1. How would you rate your personal overall experience of the TBL theme this week? | 0 | 14 (5.5%) | 55 (21.7%) | 129 (51.0%) | 55 (21.7%) | 3.9 (0.8) |
|  | **Strongly Disagree** | **Disagree** | **Neutral** | **Agree** | **Strongly Agree** | **Mean (SD)** |
| 2. The case was presented clearly and linked well with the mid-week learning activities and resources | 4 (1.8%) | 11 (5.0%) | 53 (23.9%) | 100 (45.0%) | 54 (24.3%) | 3.9 (0.9) |
| 3. The resources required for the case were made clear to me | 4 (1.8%) | 19 (8.6%) | 55 (24.8%) | 87 (39.2%) | 57 (25.7%) | 3.8 (1.0) |
| 4. The resources all seemed relevant to the session | 4 (1.8%) | 14 (6.3%) | 60 (27.0%) | 93 (41.9%) | 51 (11.7%) | 3.8 (0.9) |
| 5. The resources were of an appropriate length (didn't take too much time to review) | 12 (5.4%) | 31 (14.0%) | 47 (21.2%) | 83 (37.4%) | 49 (11.2%) | 3.6 (1.1) |
| 6. It was clear what needed to be done before the Friday session | 4 (1.8%) | 22 (9.9%) | 53 (23.9%) | 74 (33.3%) | 69 (31.1%) | 3.8 (1.0) |
| 7. The resources were easy to access and use | 4 (1.8%) | 4 (1.8%) | 38 (17.1%) | 91 (41.0%) | 85 (38.3%) | 4.1 (0.9) |
| 8. The intended learning objectives were clearly defined at the start of the week | 1 (0.5%) | 7 (3.2%) | 22 (10.0%) | 75 (34.1%) | 115 (52.3%) | 4.4 (0.8) |
| 9. The intended learning objectives were relevant to the content of the learning materials and the Friday session | 3 (1.4%) | 12 (5.5%) | 43 (19.5%) | 80 (36.4%) | 82 (37.3%) | 4.0 (1.0) |
| 10. The content of the Friday session were worked through at an appropriate pace | 8 (3.6%) | 19 (8.6%) | 43 (19.5%) | 75 (34.1%) | 75 (34.1%) | 3.9 (1.1) |
| 11. There was sufficient time available for questions and discussions | 8 (1.8%) | 34 (15.5%) | 41 (18.6%) | 67 (30.5%) | 70 (31.8%) | 3.7 (1.2) |
| 12. The Monday and Friday sessions integrated well with each other and the other materials delivered during the week | 8 (3.6%) | 10 (4.5%) | 48 (21.8%) | 91 (41.4%) | 63 (28.6%) | 3.9 (1.0) |
| 13. The sessions across the week were engaging, making attendance feel worthwhile | 8 (3.6%) | 10 (4.5%) | 60 (27.3%) | 85 (38.6%) | 57 (25.9%) | 3.8 (1.0) |
| 14. The Friday TBL session was interactive and engaging | 2 (0.9%) | 10 (4.5%) | 41 (18.6%) | 79 (35.9%) | 88 (40.0%) | 4.1 (0.9) |
| 15. My team has a good mix of students from different backgrounds, bringing in a range of different viewpoints | 3 (1.4%) | 2 (0.9%) | 16 (7.4%) | 66 (30.6%) | 129 (59.7%) | 4.5 (0.8) |
| 16. Everyone in my team participates at a sufficient level | 6 (2.8%) | 13 (6.0%) | 23 (10.6%) | 59 (27.3%) | 115 (53.2%) | 4.2 (1.0) |
| 17. There is effective communication between all of my team members | 3 (1.4%) | 9 (4.2%) | 19 (8.8%) | 69 (31.9%) | 116 (53.7%) | 4.3 (0.9) |
| 18. Disagreements within my team are resolved effectively | 1 (0.5%) | 0 | 14 (6.5%) | 54 (25.0%) | 147 (68.1%) | 4.6 (0.7) |
| 19. All team members are treated with equal respect and are given equal voice during the sessions | 2 (0.9%) | 3 (1.4%) | 10 (4.6%) | 39 (18.1%) | 162 (75.0%) | 4.7 (0.7) |
| 20. Myself or members of my team experience anxiety due to needing to work in a team environment | 95 (44.0%) | 48 (22.2%) | 26 (12.0%) | 17 (7.9%) | 30 (13.9%) | 3.7 (1.4)* |
| 21. The facilitators were knowledgeable about the topic and provided key information when it was needed | 1 (0.5%) | 10 (4.7%) | 31 (14.4%) | 75 (34.9%) | 98 (45.6%) | 4.2 (0.9) |
| 22. The experts who Zoomed in to the session were effective in answering questions raised by the students | 3 (1.4%) | 15 (7.0%) | 39 (18.1%) | 74 (34.4%) | 84 (39.1%) | 4.0 (1.0) |
| 23. The facilitators were physically available to provide us with guidance when it was needed | 1 (0.5%) | 11 (5.1%) | 29 (13.5%) | 68 (31.6%) | 106 (49.3%) | 4.2 (0.9) |
| 24. The range of teaching sessions during the week helped in learning the materials related to the TBL theme | 1 (0.5%) | 15 (7.1%) | 36 (17.0%) | 98 (46.2%) | 62 (29.2%) | 4.0 (0.9) |
| 25. I feel that I understood the overall case materials to the depth that the teaching staff expect | 3 (1.4%) | 25 (11.8%) | 61 (28.8%) | 73 (34.4%) | 50 (23.6%) | 3.7 (1.0) |
| 26. What I learned during this week is applicable to real-world situations | 2 (0.9%) | 5 (2.4%) | 29 (13.7%) | 70 (33.0%) | 106 (50.0%) | 4.3 (0.9) |

* As the question was negatively framed, the response scoring was inverted to provide a mean score comparable to the other Likert-scale question

Appendix 8. Additional Responses to Open-Ended Questions

Participant quotes are often suggested as being ‘cherry-picked’ to over-emphasise the findings in qualitative research. The quotes below are those that were best articulated and are in addition to those presented in the main text to demonstrate the broad agreement across staff and students in the survey and focus groups. These are no duplicate students represented under each bolded title below.

**Student enjoyment and engagement with learning materials and perceived clinical relevance**

*“The information we needed to learn was quite interesting and had obvious links to the clinical environment. It was also quite clear how much depth we needed to learn.”*

*“I found the lecture on sustaining life on ICU very engaging. I like how the lecturer kept challenging the students in the audience by asking questions.”*

*“Mostly the clinical relevance of what I was learning. I felt this week a lot of the anatomy and ILOs covered more practical stuff (like chest drains and anaesthetics) rather than theoretical.”*

*“It’s a realistic scenario that you might have to meet, and although it is a stressful situation you’ll have to learn to deal with these kinds of scenarios in hospital too.”*

*“The pneumothorax lecture was useful as it clearly covered an ILO and I could see the clinical relevance to the content we were learning.”*

*“Clinal applications surrounding asthma (or COPD etc) rather than just biology/physiology. Actually learning the treatments and symptoms and examinations in the CSLC was good.”*

*“I enjoyed learning more about actual conditions that affect people. The material you learn I can see myself using as a doctor.”*

*“I thought that the case and lectures were interesting and enjoyed learning about mitral valve disease, ECGs, heart anatomy.”*

*“The lectures - specifically those on blood pressure and it’s control (and how this tied into other themes like cancers and pharmacology after the Friday session).”*

*“All the different kinds of drugs to treat hypertension and which drugs to give/ to not give depending on age, ethnicity and any other underlying problems.”*

*“The lecture surrounding this case I found to be very useful, especially the one which demonstrated the outflow of blood of intravenous cannulas with different gauges.”*

**Quotes highlighting student perceived usefulness of learning material alignment/integration**

*“I found the case topic very useful and I like how the anatomy was included in the ILOs this time. It made it easier to get through them all, I found learning about the causes, symptoms, physiology, treatments and different types of pneumothoraxes really interesting.”*

*“It was interesting to learn about COPD. It was good that it followed on nicely from last week’s theme so that concepts from the previous week could be reviewed.”*

*“Diagnosing the patient in TBL application exercise (had to apply learning from different themes to get to the answer).”*

*“I liked how this week was very nicely linked to the week before and felt like a build on.”*

*“It was an exciting topic, it linked with previous knowledge so felt like it all made sense. For example there was a lot of stuff on blood pressure which was covered in the previous theme.”*

**Student perceived benefits of IRAT and AES in TBL sessions**

*“iRAT is very useful to gage the information that we need to cover and the team activities are really good for thinking in a more clinical way.”*

*“I liked discussing the different asthma drugs with my TBL. I found that it solidified my knowledge of what I need to know about them.”*

*“It was a really nice to be able to consolidate the ideas and topics that were covered this week, as well as fill in any remaining gaps in our knowledge of the theme.”*

*“I found the application exercises this week the most enjoyable, along with the consultation skills clinic, where we were able to practise both the physical examination aspect and also the speaking.”*

*“I particular enjoyed the phys/pharm on ECG as it provided insight in how to interpret one and the meaning behind different ECG patterns.”*

*“The tRAT is the best part because you get to understand why your answers are wrong/why someone put the right answer from the perspective of a fellow medical student.”*

*“I feel the IRATS gives us a very good glimpse on our studying and allow us to evaluate our studying during the week.”*

*“The iRAT and tRAT are very useful for gauging if the amount of work you have done is enough.”*

*“I enjoyed how the diagrams for the coagulation cascade were asked [during the application exercises] as well as the follow up questions I felt were relevant and something new I had learned that I did not find in the lectures.”*

*“This weeks TBL gave me more of an idea on what aspects of the case I needed to focus on or look more into. For example, compartment syndrome had not been something I researched before the session.”*

*“I was able to consolidate my knowledge on the coagulation cascade (helped me reinforce my memory), able to learn more about warfarin and its alternatives, and aspects that i revised came up in the IRAT.”*

*“Being given clinical scenarios where we had to come up with a diagnosis and recommended treatment plan with our TBL groups. This allowed me to put my week's learning into practice and fill any gaps.”*

**Timing issues related to Friday TBL sessions (IRAT, TRAT, AES)**

*“I wish there was more time to go over the questions with the tutors after doing the trat as it's usually very rushed and we always end up being cut off.”*

*“We don't have enough time to discuss with the tutors the answers to the questions and to fully understand them. I find this part of TBL sessions the most helpful, but it is too quick to be productive.”*

*“For some of the questions we didn’t have time to fully discuss the answers with the tutors so I did not understand a few points.”*

*“In the Friday TBL session there wasn't enough time for group discussion and the facilitators were often cut off by moving on to the next task.”*

*“We didn’t get enough time to discuss our answers with the rest of the class and that means we don’t know whether out answers are correct or not for the application exercises.”*

*“Not enough time to go through the IRAT questions and application exercises. Think this varies with each room as last semester there might've been more time, whereas this semester there seems to be less time.”*

*“We need more time. We always start discussions but never finish them! We also discuss with tutors when we are done with activities but never finish the discussion! How will I know my answers are right?”*

*“A few of the difficult TRAT questions weren't answered. On a weekly basis, it feels as though there isn't enough time allocated to allow tutors to go through the TRAT answers.”*

*“We need more time for whole room discussion after trats and application exercises as questions do not get answered due to lack of time.”*

*“Perhaps, more time explaining the answers of the TRAT by the teachers / advisors would be better because sometimes the students don't know the answer.”*

**Timing issues and requesting more questions answered by the expert panel**

*“I think the expert panel needed to manage their time slightly better as there were some questions that weren't answered”*

*“Need more time with Expert Panel and to ask questions that arise from later activities not just the TRAT”*

*“Sometimes the expert panel don't manage to answer all the burning questions.”*

*“Some questions we had, were perhaps pushed back to the expert panel which might have not had enough time to explain”*

**Lack of alignment between lectures, ILOs and IRAT**

*“Some lecture content not in ILOs and some ILOs not in lectures, bioscience resource etc. more clarity on learning objectives needed”*

*“It would be useful if the lectures said specifically which ILO's they cover so that we know whether to do more research or if that is the level of knowledge we need”*

*“Although the learning materials covered our pharmacology ILO's, we found that the TRAT included questions about receptors that weren't mentioned in the resources.”*

*“Large number of ILOs were not connected to a lecture/mini learning session/provided resources. Made this week’s aims unclear”*

*“Anatomy this week was not relevant to TBL case. A lot of the ILOs were not covered in teaching sessions and required individual input.”*

*“Many of the questions on IRAT were not related to the lectures this week hence many of us found it very difficult”*

*“There was an activity in the TBL session about intracellular and extra cellular fluid but this wasn’t really covered in the week’s learning. The ILOs didn’t seem to match with the TBL content as much.”*

*“The lectures for this week could've focused on the ILOs more and answered them better.”*

Appendix 9. Comparison of financial elements of PBL and TBL for one academic year.

| Costed Item | PBL | TBL | Difference |
| --- | --- | --- | --- |
| Academic Staff Time*  Opening session  Closing session  Total | £25 701  £42 836  £68 537 | £2 705  £43 286  £45 991 | -£22 996  £450  -£22 546 |
| Technology Costs^ | £0 | £10 023 | £10 023 |
| e-Learning Support Staff | £0 | £7 435 | £7 435 |
| Total Cost Difference | £59 970 | £63 450 | -£5 088 |

The values listed relate to one full academic year for a student cohort of 450 students.

* Combined value of staff preparation and facilitation time, assuming all academics are ‘Level 7’ lecturers at the University of Manchester for the 2023/2024 academic year

^ Combined value of the LAMS online learning management system, laptops, Bluetooth speakers, and other materials for Friday ‘closing’ videoconferencing (including expert panel)
